# Intrauterine Zika Virus Infection: An Overview of the Current Findings

**DOI:** 10.3390/jpm15030098

**Published:** 2025-03-01

**Authors:** Ana Luiza Soares dos Santos, Beatriz Bussi Rosolen, Fernanda Curvelo Ferreira, Isabella Samões Chiancone, Stefany Silva Pereira, Karina Felippe Monezi Pontes, Evelyn Traina, Heron Werner, Roberta Granese, Edward Araujo Júnior

**Affiliations:** 1 Discipline of Woman Health, Municipal University of São Caetano do Sul (USCS), São Caetano do Sul 09521-160, SP, Brazil; ana.santos4821@uscsonline.com.br (A.L.S.d.S.); beatriz.rosolen@uscsonline.com.br (B.B.R.); fernanda.ferreira@uscsonline.com.br (F.C.F.); isabella.chiancone@uscsonline.com.br (I.S.C.); stefany.pereira@uscsonline.com.br (S.S.P.); araujojred@terra.com.br (E.A.J.); 2 Department of Obstetrics, Paulista School of Medicine, Federal University of São Paulo (EPM-UNIFESP), São Paulo 04023-062, SP, Brazil; karina.pontes@unifesp.br (K.F.M.P.); etraina@unifesp.br (E.T.); 3 Service of Gynecology and Obstetrics, Ipiranga Hospital, São Paulo 04262-000, SP, Brazil; 4 Department of Fetal Medicine, Biodesign Laboratory DASA/PUC, Rio de Janeiro 22453-900, RJ, Brazil; heron.werner@gmail.com; 5 Department of Biomedical and Dental Sciences and Morphofunctional Imaging, “G. Martino” University Hospital, 98100 Messina, Italy

**Keywords:** Zika virus, pregnancy, intrauterine infection, imaging diagnostic methods, fetal malformations, vertical infectious disease transmission

## Abstract

Zika virus (ZIKV) is a mosquito-borne flavivirus of the family Flaviviridae. The association between ZIKV and microcephaly was first described in Brazil in 2015. The risk of vertical transmission occurs in pregnant women with or without symptoms, and the risk of malformation appears to be worse when infection occurs in the first and second trimesters of pregnancy. The rate of vertical transmission varies from 26 to 65%, and not all fetuses develop malformations. The incidence of malformations resulting from transmission is uncertain, ranging from 6–8% in the US to 40% in Brazil. Congenital ZIKV syndrome is a set of clinical manifestations that can affect the fetus of a mother infected with ZIKV. The manifestations are broad and nonspecific, including microcephaly, subcortical calcifications, ocular changes, congenital contractures, early hypertension, and pyramidal and extrapyramidal signs. Other findings such as growth restriction and fetal miscarriage/death may also occur. Our aim in this article is to review the literature on mosquito transmission, clinical presentation, serologic diagnosis, intrauterine transmission, pre- and postnatal imaging diagnostic findings, and short- and long-term follow-up.

## 1. Introduction

Zika virus (ZIKV) belongs to the family Flaviviridae and the genus Orthoflavivirus. The first human infection was confirmed in Uganda in 1962–1963, but evidence of infection was found as early as 1954. Historically, the virus was first isolated in East Africa in 1947 in the blood of a sentinel monkey in the Zika Forest in Uganda. After the initial infection, epidemiologic studies suggest that the geographic distribution of the virus shifted toward sub-Saharan Africa and Southeast Asia. The historical milestone of the disease occurred in 2007, with the first confirmed outbreak on the island of Yap [1]. The virus was introduced into Brazil from the Pacific Islands between 2013 and 2015 and rapidly spread throughout the Americas. As such, it became the first and largest infectious disease linked to birth defects discovered in more than half a century [2].

For 70 years, ZIKV infection was rarely recognized and studied because the disease could have been misdiagnosed as dengue virus (DENV) infection based on clinical symptoms and laboratory diagnosis due to serologic cross-reactivity and similar clinical presentation [1,3]. In addition, ZIKV infection went unnoticed due to the absence of major outbreaks during the same period and its silent nature concerning the mode of transmission [1]. ZIKV has two modes of transmission, including the sylvatic, which is associated with non-human primates and forest-dwelling *Aedes* mosquito species [3], mainly belonging to the subgenera *Stegomyia* and *Diceromya*, including *Aedes africanus*, *Aedes luteocephalus*, *Aedes furcifer*, and *Aedes taylori* in Africa and Asia. However, the sylvatic transmission cycle has not been found in the Asian environment. In the urban environment, ZIKV has a human–mosquito–human transmission cycle in which species in the *Aedes* subgenus *Stegomyia*—*Aedes aegypti* and *Aedes albopictus*—have been associated with almost all epidemics, including those in the Americas [2].

After the discovery of the first modes of transmission, new forms of infection, the non-vectorial ones, were found, indicating that ZIKV can be transmitted from mother to fetus during pregnancy, sexually, by blood transfusion, and through fluids [2]. Maternal–fetal transmission has been largely responsible for severe clinical manifestations, such as microcephaly and other congenital malformations, around the world, having been first reported in French Polynesia in 2013. This case was confirmed by the presence of viral RNA detected in the amniotic fluid of pregnant women with symptoms of ZIKV [1], as well as its presence in brain tissue and the placenta [2] and spontaneous miscarriage, confirming the theory of maternal–fetal transmission [1]. In 2016, more than 4300 cases of microcephaly were detected in Brazil, particularly in the state of Pernambuco. The previous year, there was a correlation between the state where the first ZIKV infections were reported in the first trimester of pregnancy [4] and the number of newborns with microcephaly [2], including lissencephaly, cerebral calcifications, and cerebral atrophy, detected by different imaging diagnostic modalities such as trans fontanelle ultrasound, magnetic resonance imaging (MRI), and computed tomography (CT). MRI was superior to trans fontanelle ultrasound in defining brain pathology, showing pachygyria, reduced gyrification, thin parenchyma, and asymmetric ventriculomegaly, especially in the posterior horns and colpocephaly, also associated with dysgenesis of the corpus callosum. Nevertheless, cerebral calcifications were detected on trans fontanelle ultrasound and were not observed in MRI [4].

Infection by ZIKV during pregnancy is asymptomatic in 80% of cases; the incubation period varies from 3 to 40 days [5], and symptoms, if present, last an average of 4 days [2]. Among the major manifestations, the most common symptoms are maculopapular rashes, fever, arthralgia, non-purulent conjunctivitis, headache, edema, vomiting [4], and retro-orbital pain [2], and the disease is self-limiting. It is important to note that symptomatic congenital infection will not occur in all exposed fetuses. Therefore, transmission can be prevented by practicing safe sex with condoms and preventing mosquito bites by using insect repellents and mosquito nets [1].

The diagnosis of ZIKV infection presents a several challenges [6], and screening is performed using two main approaches: (1) the detection of viral RNA by nucleic acid amplification testing (NAAT) using real-time quantitative reverse transcription-polymerase chain reaction (RT-PCR), and (2) the detection of antibodies (IgM and IgG) using an enzyme-linked immunosorbent assay (ELISA), which should be confirmed by microneutralization or plaque reduction neutralization (PRNT) assays [5] after one week with specific titers for ZIKV appearing more than four times, or by seroconversion, in which there is an exchange of IgM from the initial infection for IgG between samples collected one week apart [7]. The main difficulty in the diagnosis of ZIKV infection is the immunological cross-reaction with other viruses of the flavivirus family, such as DENV, yellow fever, and others. Therefore, false-positive or uninterpretable results may be obtained in the cases of vaccination or current or past infection with other arboviruses [6], and for this reason, there is an important correlation between the laboratory change and the clinical condition of the patient [7]. Molecular tests based on serum and urine are the most recommended [6], with better sensitivity when carried out at the onset of symptoms between 3 and 14 days [5]. New studies show the effectiveness of saliva collection, but other studies show controversy regarding its benefits [6].

Pregnant women with a suspected or confirmed ZIKV infection should undergo serial prenatal ultrasound scans to assess fetal anatomy, particularly the central nervous system (CNS), amniotic fluid volume, and fetal growth. To improve morphologic analysis, MRI is recommended at 30 weeks to better assess cortical development [5]. Amniocentesis should not be performed routinely because ZIKV RNA can be transient, so the decision to perform the procedure in the cases of maternal exposure and fetal changes should be discussed with the patient. There is no specific treatment for ZIKV using antivirals, so the approach is based on palliative treatment along with symptomatic treatment [5].

After birth, the extent of clinical manifestations resulting from intrauterine ZIKV infection is variable and requires further research to determine the extent of neurological impairment. In most cases, head circumference may be normal, but long-term cognitive, adaptive, and behavioral abnormalities may be present. Among the most common conditions observed are low birth weight, language delay, especially receptive language, sensorineural deafness, and, in general, delays in neuropsychomotor development, demonstrating the importance and need for a multidisciplinary team to monitor the effects in childhood [8].

## 2. Etiology

ZIKV consists of a single strand of non-segmented positive-sense RNA belonging to the Flaviviridae family [9,10,11,12,13,14]. The genome is approximately 40–60 nm in diameter and 11 kb in size, and the open reading frame is 5′-C-prM-E-NS1-NS2A-NS2B-NS3-NS4A-NS4B-NS5-3′ [15,16]. After cleavage by the host’s cellular glycosyltransferases, the translated polypeptide produces 10 proteins, three of which are structural; capsid (C), envelope (E), and membrane (M) or membrane precursor (prM), and the other seven are non-structural, NS1, NS2A, NS2B, NS3, NS4A, NS4B, and NS5, which are responsible for processing, replication, and the assembly of new viral models [9,10,15,17,18,19,20,21,22,23,24].

The structural proteins have the following functions: protein C facilitates the packaging of viral particles, protein M induces protein E rearrangement and facilitates the maturation of virions, and finally, protein E is directly related to the binding to host cells and recognition by the immune system [16,17,25,26]. Among the non-structural proteins, NS1 and NS4 are post-translationally cleaved [17,21]. This means that NS1 is directly linked to viral replication and immune evasion, as it is responsible for conserving the viral protein in monomeric (cytoplasmic), dimeric (membrane protein ligand) and hexameric (secretory protein, known as sNS1) forms [17,27,28,29,30]. Together with NS1, NS2 plays an important role in viral replication and the evasion of the host immune system [17,31]. NS3 is an RNA helicase that is directly associated with two other non-structural proteins, NS2B and NS4A [17,19,32]. The NS4A and NS4B proteins have been implicated in blocking neurogenesis and promoting autophagy [17,33]. Finally, NS5 is an RNA polymerase-dependent methyltransferase involved in the synthesis and modification of viral RNA [17,19].

## 3. Epidemiology

ZIKV was first identified in 1947 in a rhesus monkey in the Kampala and Uganda regions of the African continent [10,34,35,36,37,38,39,40] and isolated in 1948 in *A. africanus* mosquitoes in the same area [16,41]. Five years after the virus was isolated [10,41], the African continent experienced its first ZIKV epidemic [10,40,41,42,43]. Following the confirmed cases of ZIKV infection in humans, epidemiologic surveillance on the subject increased, leading to the conclusion that ZIKV was distributed in countries in Africa—African strain—and Asia—classic Asian strain [16,40,44].

Furthermore, with the wider scope of studies, countries in Asia such as India, Indonesia, Malaysia, Pakistan, Philippines, Thailand, and Vietnam reported cases of ZIKV between the years 1950 and 1980 [16,45,46,47,48,49]. After six decades, ZIKV was reported outside the African and Asian continents, affecting approximately three quarters of the population of the Yap Islands, located in the Confederated States of Micronesia in the western Pacific Ocean, in 2007 [16,37]. The strain that caused this outbreak was identified as the classic Asian strain [11,16]. Six years later, French Polynesia was affected, resulting in 30,000 confirmed cases. However, due to asymptomatic cases, it is estimated that the number of infected individuals was underestimated [16,50,51]. In subsequent years, other Pacific islands, including Easter Island, the Solomon Islands, the Cook Islands, and New Caledonia, experienced peaks of contamination [16,52,53,54,55].

**Figure 1:**
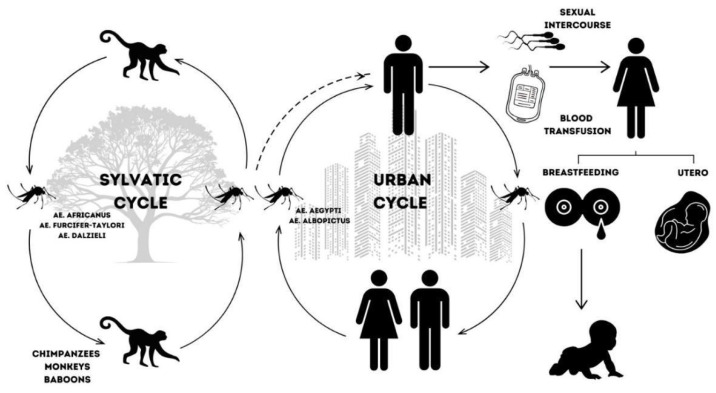
Zika virus sylvatic and urban transmission cycles.

Between 2013 and 2015, ZIKV swept through the Americas. However, Brazil recorded its first case in the early months of 2015 in the state of Rio Grande do Norte [16,56]. The virus spread rapidly, reaching pandemic proportions in May 2015 [10,57,58]. In the same year, autochthonous transmission was detected in 18 other Brazilian states [16,51,59]. Following the Brazilian outbreak, the World Health Organization (WHO) declared ZIKV a public health problem [10,36,60] and reported on the importance of reducing transmission rates and the need of additional care for pregnant women and women of reproductive age [10,57,61,62]. Figure 1 shows the ZIKV sylvatic and urban transmission cycles.

### 3.1. Vectorial Transmission

The transmission cycle of Zika virus (ZIKV) involves arthropods belonging to the *Aedes* genus: *Aedes aegypti*, *Aedes albopictus*, *Aedes hensili*, and *Aedes polynesiensis* [17,63,64,65,66], as well as the domesticated mosquito *Culex quinquefasciatus* [10,22,67], rhesus monkeys [10,34,35,36,37,38,39,40], and humans. The enzootic cycle of the disease occurs between mosquitoes and primates, including monkeys, chimpanzees, and baboons. When arthropods infected with the virus bite humans, whether in sylvatic environments or urban areas due to migration, the urban cycle of the disease is initiated [10,68,69,70,71].

### 3.2. Sexual Transmission

In 2008, the United States was the first country to report a case of sexually acquired ZIKV infection [17,72]. However, it was not until 2013 that the virus was isolated in semen [17,73]. Studies in Tahiti have shown that ZIKV can persist in semen for 412 days after the remission of ZIKV symptoms [17,74]. Research in immunodeficient mice has shown that the virus infects Sertoli cells, Leydig cells, spermatogonia, primary spermatocytes, and luminal epithelial cells of the male reproductive system. Thus, it is concluded that ZIKV can infect somatic and germ cells, elucidating the means of transmission from mother to fetus [9,75,76]. Among the cases described in the literature, the transmission of ZIKV between men and women is approximately 94% [10,72,77].

### 3.3. Vertical Transmission

During pregnancy, the immune system is modulated to protect the fetus, with the formation of tolerogenic dendritic cells, reduced monocyte and macrophage activity, and decreased recruitment of natural killer (NK) cells and T and B lymphocytes [9,78,79,80]. This modulation may help ZIKV to penetrate the placental barrier and infect cytotrophoblasts, syncytiotrophoblasts, and decidual and mesenchymal cells [9,81,82,83,84,85]. ZIKV also has an enhanced ability to infect the fetus due to its tropism for placental cells and reproductive tissues, which is associated with increased expression of receptors such as AXL, DC-SING, and TIM-1 in these tissues and factors associated with the host organism [9,86].

ZIKV has been isolated in samples of amniotic and cerebral fluid from newborns. This suggests that the virus crosses the placental barrier and reaches the fetus. The first record of transmission during pregnancy occurred in French Polynesia, where serum samples from mothers and their newborns tested positive 4 days after delivery in the 2013–2014 outbreak [10,17,56,58,87,88]. Cases of postpartum transmission through breastfeeding have also been reported [17,89,90]. However, studies have reported that newborns breastfed by ZIKV-positive mothers remained asymptomatic due to the immunologic properties associated with breast milk [91,92].

### 3.4. Other Transmission Forms

Samples of blood, saliva [17,93,94,95], and urine [10,96,97] from infected patients have been found to contain ZIKV. In 2016, Brazil recorded cases of transmission via blood transfusion, made through blood donors who were asymptomatic for the disease [10,98]. Brazil is the country with the most cases of ZIKV infection via blood transfusion [10,99]. Studies have shown the presence of viable ZIKV in the bloodstream after 2 months of infection [10,100]. ZIKV was detected in infectious titers in human urine cultures about a month after the onset of symptoms [10,96,97,100]. In addition to the presence of ZIKV in urine, the larvae and pupae of *Aedes aegypti* and *Aedes albopictus* matured in water contaminated with ZIKV can acquire the virus and become infected adult females [97].

## 4. Physiopathology

The reproductive cycle of ZIKV in the Flaviviridae family is similar and involves the process of autophagy, which promotes intracellular infection and increased viral replication [9,16,101]. Thus, ZIKV infects humans through viral projections that bind to cell receptors [16,101], and the viral response depends on activation of the host’s innate and adaptive immune systems. ZIKV infection is recognized by host pattern recognition receptors (PRRs), such as Toll-like receptors, which lead to increased expression of type 1 interferon (IFN-1), which is important in reducing viral spread to other surrounding cells. After signaling by the intrinsic immune system, host cells begin to defend themselves through autophagy, apoptosis, and RNA interference [9].

Once the viral membrane has fused to the membrane receptors, facilitated by viral glycoprotein E, the virus can be endocytosed. Upon contact with the endosomal lumen, the virus acidifies it, inducing structural changes in the membrane glycoproteins capable of releasing the viral genome protected by the capsid [101]. Autophagy mediated by the viral system and intracellular degradation via lysosomes contribute to the increased viral replication. To enable autophagy, the NS4A and NS4B proteins inhibit the AKT-mTOR complex, formed by the binding of AKT kinase and the mTORC1 complex, in human fetal neural stem cells (fNSCs). The viral protein NS4A binds to the mitochondrial membrane and activates the Bcl-1-associated X protein (BAX), forming the Bcl-2 complex, which interacts with the voltage-dependent mitochondrial anion channel, triggering membrane potential loss and, consequently, the release of cytochrome c. This process leads to the activation of caspase 9 and caspase 3, demonstrating the activation of the intrinsic apoptosis pathway [9].

After release into the endosome, the genome is translated into a single positive-sense polypeptide that is replicated via a negative-sense RNA intermediate and processed to produce new viral proteins [102]. In addition, viral protein C helps package the new RNA and the newly formed virions, which are then secreted into the cytoplasm as enveloped viruses. The mature virions are released into the extracellular environment by exocytosis after the E and prM proteins have undergone a translation process [25].

Another host defense is the production of pro-inflammatory cytokines and signaling via IFN-1. As the concentration of interferons increases, there is an increase in the recruitment of IFN-stimulated genes (ISGs), the final effectors of the response to infection and important for reducing viral spread to other cells [103,104]. Toll-like receptors (TLRs) are present on immune and epithelial cells, and their main function is to identify viral pathogens present in the host and induce the production of IFN-1, thereby reducing the viral load. The Toll-like receptor 3 (TRL3), present on myeloid dendritic cells [9], skin fibroblasts, and keratinocytes [86], induces the production of the INF-β inducing adapter (TRIF) containing the TIR domain, which is used to recruit tumor necrosis factor (TNF) and intermediate factors to produce IFN-1, an inducer of autophagy, apoptosis, and RNA interference [105]. In addition, INF-I is a product of the cyclic guanosine monophosphate (GMP)–adenosine monophosphate (AMP) stimulator of INF genes (cGAS-STING) pathway. The pathway begins with the production of GMP-APM by cGAS, which binds to the endoplasmic reticulum adaptor protein STING, activating TBK1 and IRF3, leading to the production of INF-1. The signaling pathway can be initiated by the cGAS system after identification of its own or a foreign genome [9,106].

Studies in pregnant mice have shown that INF-1 activation is detrimental to fetal development because it induces cell death in virus-infected endothelial cells and placental cells, impairing placental development and thus the maternal–fetal barrier [107,108]. ZIKV entry occurs in a variety of cellular tissues due to the virus’ ability to evade the host immune system and reach trophoblast cells, skin fibroblasts, umbilical cord endothelial cells, and cytotrophoblast cells [9]. As noted above, inhibition of full AKT-mTOR by the viral proteins NS4A and NS4B triggers signaling to cells in the fetal central nervous system, thereby blocking PI3K activity and allowing post-translational conformational changes in AKT. The importance of complete PI3K-AKT in fetal brain development has been highlighted [9]. Another role of NS4B is cell death, which is triggered by the accumulation of the viral protein in the outer membrane of the mitochondria, increasing membrane permeability and allowing the release of factors such as cytochrome C, which activates caspases 3 and 9 [9,109].

The relationship between ZIKV and maternal–fetal transmission, which is responsible for the cases of microcephaly in the 2015 outbreak in Brazil [110], is explained by the fact that in vitro ZIKV infects neuroprogenitor cells derived from human induced pluripotent stem cells (hNPCs) of the fetus [16,111,112,113]. When infected, the cells exhibit ZIKV-induced cytopathic effects, such as the induction of cell death, leading to abnormalities in fetal brain formation [113]. Infection of pluripotent stem cells results in the activation of innate immune pathways through TLR3 and other ISGs and a reduction in the expression of genes associated with neurogenesis [114]. In addition, hNPCs are reported to induce the development of neurospheres and cerebral organoids, which are responsible for initial neurogenesis in the first trimester of pregnancy. Thus, when hNPCs are infected, there is a reduction in the number of neurospheres and cerebral organoids in the early stages of neurogenesis [16,112,113].

## 5. Fetal Malformations

ZIKV has a tropism for cells in the CNS, which explains most of the changes in this system in infected fetuses and newborns [115]. The expression of the disease is directly related to the gestational age at which the maternal infection occurred, with infections in the first and second trimesters being more deleterious than those in the third trimester [115,116,117,118]. As a result of exposure through vertical transmission of ZIKV, fetuses have a 5–14% risk of developing congenital ZIKV syndrome, of which 80% have microcephaly [119].

Microcephaly can be classified as primary, which occurs before birth as a result of alterations in the brain development process due to the infection established during the fetal period, and secondary, in which the fetus does not show structural alterations, and the malformations become noticeable in the postnatal period due to mutations in the maturation of neurons. It may also be proportional, in the case of a small-for-gestational age (SGA) fetus, or disproportionate, in which the head circumference does not match the other anthropometric measurements [117,120]. Other prenatal imaging findings associated with central nervous system abnormalities have been reported, such as ventriculomegaly (33%), intracranial calcifications (27%), lissencephaly, delayed myelination, cerebral atrophy, cortical malformations, hypoplasia of the corpus callosum and cerebellum, as well as hydrocephalus [115,116,117,120]. Ventriculomegaly can be classified based on the degree of ventricular dilation as mild (10 to 12 mm), moderate (13 to 15 mm), or severe (>15 mm) [115]. The formation of folds in the scalp in the occipital region indicates restriction of brain growth [117].

**Figure 2:**
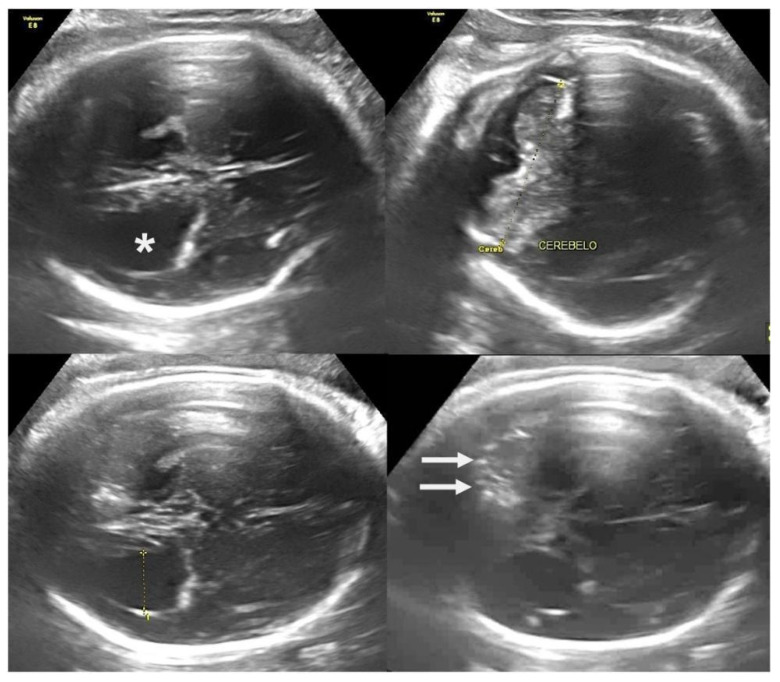
Prenatal ultrasound (37 weeks) axial view findings showing periventricular calcifications (arrows) and ventricular dilatation (*). Note normal cerebellum.

**Figure 3:**
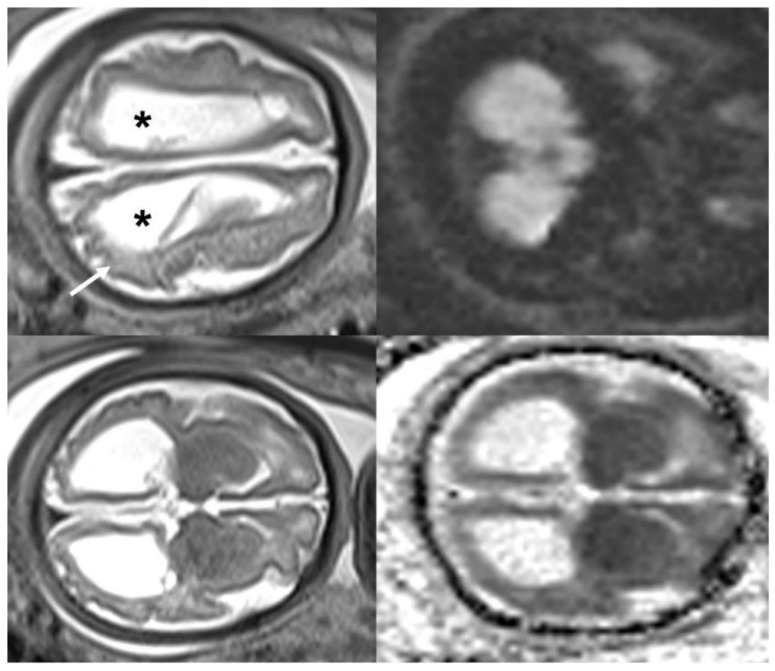
Magnetic resonance imaging axial T2-weighted and diffusion-weighted magnetic resonance imaging at 37 weeks, showing ventricular dilatation (*) and smoothness of the brain surface (arrow). Note normal cerebellum.

**Figure 4:**
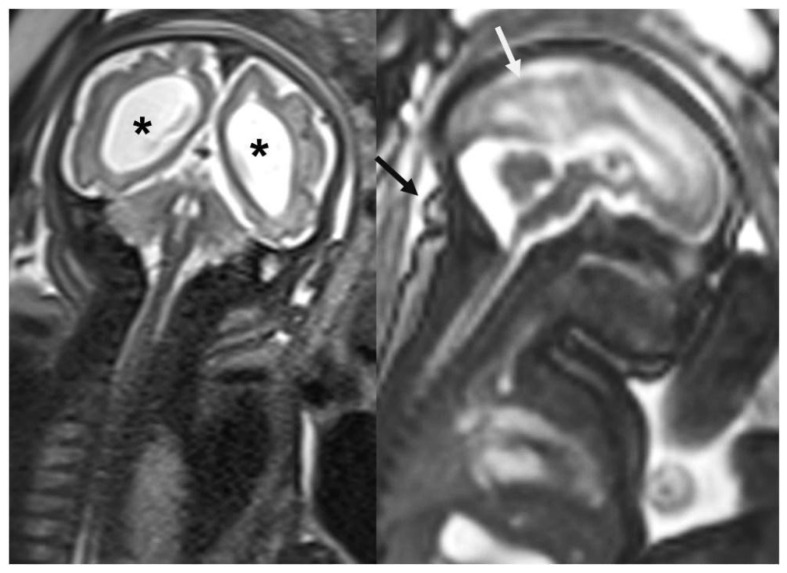
Coronal and sagittal plane (T2-weighted magnetic resonance imaging) showing smoothness of the brain surface (white arrow). Note microcephaly and redundant skin fold (black arrow). Ventricular dilatation is clearly seen in the coronal plane (*).

Studies have showed that the fetal mortality rate in Brazilian pregnant women infected with ZIKV is 7% [117]. Other adverse perinatal outcomes such as hydrops, growth restriction, low birth weight, and preterm birth have also been reported, with preterm birth being a questionable event in some studies [116,117,118]. Recent evidence suggests that during the Brazilian epidemic, there was an increase in preterm births to avoid vertical transmission [117], challenging the hypothesis defended by other authors that ZIKV is a direct risk factor for prematurity [115]. Figure 2, Figure 3 and Figure 4 show the main fetal CNS abnormalities of intrauterine ZIKV infection.

## 6. Diagnosis

### 6.1. Maternal Diagnosis

Diagnosing ZIKV can be challenging since the symptoms and clinical manifestations of this infection could be similar to those caused by other flaviviruses, such as dengue and chikungunya [17]. Thus, in the endemic areas, such as South and Central America, the Caribbean and some parts of the African continent, when no other diseases with a similar clinical presentation are being considered, such as dengue and chikungunya, the diagnosis may be mostly clinical [121]. The RT-PCR test is considered the gold standard for confirming the diagnosis of ZIKV [17] and can be performed on samples of blood, saliva, urine, and even amniotic fluid, but it is only identified in blood and saliva in patients with up to 7 days of symptoms, after which urine or semen samples should be used [121].

In terms of clinical diagnosis, most cases are asymptomatic. If symptoms are present, the most common are low-grade fever, malaise, headache, myalgia, conjunctivitis, and pruritic rash [121]. The clinical manifestations caused by ZIKV are generally milder than those caused by other flaviviruses [122], and the illness is usually self-limiting, lasting about 7 days [121]. However, care should be taken not to underestimate the more common possible neurological sequelae, such as meningitis and Guillain–Barré syndrome, which are justified by the remarkable neurotropism of ZIKV [122].

### 6.2. Intrauterine Diagnosis

It is essential to perform imaging diagnostic examinations during pregnancy because once microcephaly is identified, the investigation of the cause must begin with these examinations. Among the possible causes, ZIKV infection should be considered [123]. Amniocentesis and subsequent analysis of the pregnant woman’s amniotic fluid aims to detect the presence of viral RNA using the RT-PCR technique [124]. Analysis of the amniotic fluid by RT-PCR is the only way to identify ZIKV in utero and thus confirm the diagnosis of fetal infection [125]. Thus, performing amniocentesis in pregnant women is essential to make a diagnosis during pregnancy in the presence of fetal abnormalities suggestive of infection, reinforcing the importance of imaging studies [125].

### 6.3. Imaging Diagnostic Methods

The main neuroimaging diagnostic methods used in the evaluation of congenital ZIKV syndrome, CT and MRI, have common findings; the main ones being a decrease in skull diameter and brain volume, i.e., microcephaly, and intracranial calcifications. Microcephaly is defined as a congenital malformation in which there is inadequate development of the brain. This malformation is found in newborns with a head circumference at birth < 2 standard deviations to that expected based on the newborn’s gestational age and sex [126,127]. It can have genetic causes, infectious causes (e.g., rubella, cytomegalovirus), or causes secondary to alcohol and drug intoxication [121]. Newborns with this condition have significant neuropsychomotor and cognitive delay [121,127].

**Figure 5:**
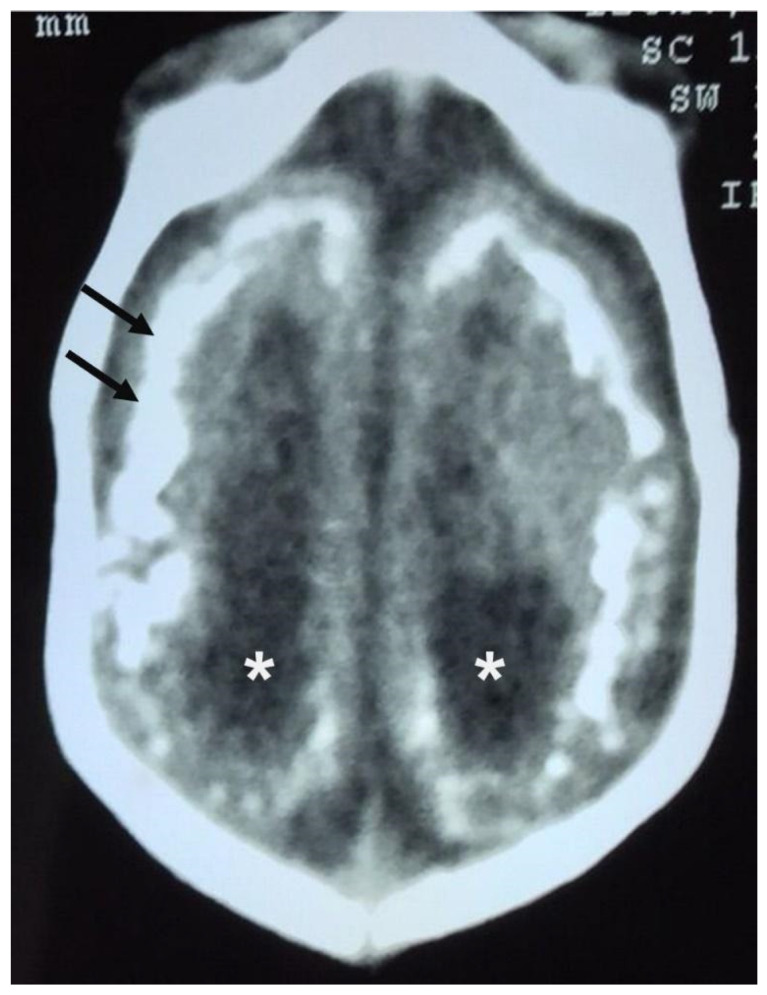
Postnatal computed tomography scan showing parenchymal atrophy, microcephaly, widespread/multiple brain calcifications (arrows), and ventricular dilatation (*).

MRI is known to be the best imaging diagnostic examination for detecting unusual skull development, such as cortical abnormalities, usually diffuse, predominantly in the frontal lobes, and even an increase in the extra-axial cerebrospinal fluid space, a consequence of impaired cerebrospinal fluid reabsorption due to the decrease in brain volume. In addition, it is common for the skull cap to have everted sutures and prominent occipital bones, leading to a “collapsed” appearance, which is explained by the continued growth of the skull even with brain reduction. CT is the most sensitive imaging diagnostic method for identifying cerebral calcifications. Studies comparing the results of CT scans of the skull of newborns affected by ZIKV congenital infection have mainly reported a reduction in the volume of the encephalic parenchyma and calcifications, both punctate and coarse in the CNS; among the main sites are the subcortical cortical junction, the basal ganglia, thalamus, and brainstem. Ventriculomegaly and colpocephaly are also found, as well as cerebellar hypoplasia and prominence of the occipital bone. Table 1 shows the main CNS abnormality findings in newborns with ZIKV intrauterine infection by CT and MRI. Figure 5 shows the CT scan findings of the CNS of a child exposed to intrauterine ZIKV infection.

## 7. Intrauterine Transmission

Studies have already identified ZIKV in placental macrophages, which are therefore considered to be the target of viral infection, leading to an exacerbated inflammatory response capable of compromising placental integrity and facilitating access to the fetus [128]. As ZIKV has significant neurotropism, vertical transmission is a concern due to its ability to affect structures of the fetal CNS. The syncytiotrophoblast, the outermost layer of the trophoblast, is highly resistant to infection by several agents, including ZIKV [123]. Explants of cells from the cytotrophoblast, the innermost layer of the trophoblast, when isolated from placentas of first-, mid-, and late-trimester pregnancies, were found to be susceptible to ZIKV; however, the syncytiotrophoblast was spared from infection [122]. Therefore, it can be concluded that ZIKV must use a number of mechanisms to enter the chorionic villi [123].

The mechanism that makes the syncytiotrophoblast resistant to ZIKV and other infections remains unclear due to the challenges of studying trophoblasts at different stages of gestation. However, this resistance is believed to be linked to its physical properties, including the high density of microvilli and their branching structures, as well as the presence of actin on its surface, which helps block viral invasion [129]. When amniotic cells from mid and late pregnancies are compared, it can be seen that mid pregnancies have higher viral titers, which corroborates the conclusion that the trophoblast is more sensitive to infection during early pregnancy and progressively increases its resistance as the syncytiotrophoblast forms [129].

One possible alternative for ZIKV to enter the placenta and cause fetal infection is through the extravillous trophoblast, trophoblast cells that invade the endometrial tissue to ensure placental anchorage and are directly juxtaposed to maternal immune cells, which, unlike the syncytiotrophoblast, are not resistant to infection, enabling viral replication in their cells and allowing the virus to reach the placental macrophages. Once it reaches the placental macrophages, called Hofbauer cells, ZIKV is able to replicate and induce an inflammatory response mediated by INF-1 and the production of pro-inflammatory cytokines, which help to damage placental integrity and, as well as impairing the supply of nutrients, facilitate viral access to the fetus [128].

## 8. Differential Diagnosis

ZIKV is an arbovirus of epidemiologic importance because of the epidemic it causes and the consequent congenital ZIKV syndrome; for this reason, it is essential to distinguish it from other diseases that also cause congenital infections of the CNS with similar adverse outcomes, such as microcephaly, and therefore, the diagnostic challenge remains due to the clinical similarity and the presence of overlapping symptoms between congenital infections [130]. An important link has been established by an imaging study of microcephaly and defects in brain organogenesis, particularly neuronal migration, indicating that the finding of cerebral calcifications is a strong indication of congenital infection of the CNS by viral agents, and when the subcortical region predominates, this variant should alert us to the diagnostic hypothesis of ZIKV [131]. Table 2 shows the main prenatal differential diagnoses of ZIKV and other intrauterine infections.

Thus, when comparing the intrauterine infections, it is possible to see that most of the increased risk of congenital infections is due to infection in the first trimester, in which CNS alterations predominate, especially microcephaly [2], with emphasis on brain calcifications, which show slightly different patterns between the infections. Comparing the ultrasound findings with an important differential in ZIKV, which may or may not present with microcephaly, and the other systems, they generally present without abnormalities and/or with non-specific changes, demonstrating a challenge in diagnosing intrauterine infections.

## 9. Postnatal Findings

Newborns exposed to ZIKV in the prenatal period have various systemic changes. In the case of CNS malformations, which are the most relevant changes, infants may suffer from seizures, restless movements, hypertonia, hypotonia, irritability, and hyperreflexia [117,118]. Despite this, a 2022 meta-analysis reported that approximately 50% of newborns infected with ZIKV during pregnancy are asymptomatic at birth [119]. This finding underscores the importance of the thorough and continuous monitoring of exposed infants, as early detection of neurological alterations allows for timely intervention, improving the child’s quality of life and development [119].

Neurosensory alterations have been shown to be significant in several studies, with the ocular and auditory systems being the most commonly cited. In terms of hearing, the main alteration found is sensorineural hearing loss, which is mainly present in children with microcephaly [115,116]. Ocular abnormalities include anterior and posterior segment changes, microcornea, microphthalmia, circumscribed chorioretinal atrophy, retinal dysplasia, abnormalities of extrinsic ocular motility and visual function, focal pigment spots, glaucoma, and hypoplasia and excavation of the optic nerve [115,117,118]. These findings are present in 30% of newborns with microcephaly, 50% of whom have nystagmus [115].

It is possible to establish a direct link between neurosensory alterations and delayed neurodevelopment of the child, as vision and hearing are fundamental elements for the newborn’s interaction with the environment. A prospective cohort study showed that 31.5% of Brazilian children infected prenatally with ZIKV had subnormal neurodevelopment and/or visual and hearing abnormalities by the third year of life [117]. Other findings of neurodevelopmental delay include speech delay, severe motor dysfunction, feeding difficulties, swallowing dysfunction, and sleep disturbances [117,118]. Arthrogryposis and clubfoot are manifestations that support the motor difficulties in children with congenital ZIKV syndrome. The deficit in gross motor development is associated with cerebral palsy, while fine motor development is associated with a lack of social interaction with the environment [115,117]. One study shows that 50% of mothers of newborns with congenital ZIKV syndrome have difficulty breastfeeding due to inadequate sucking capacity and orofacial muscles. This results in early weaning, with less than 20% of children continuing breastfeeding for 12 months [120]. These data highlight the need for follow-up and information for mothers with breastfeeding difficulties, given the importance and benefits of breastfeeding during this period for both mother and child. Dysphagia has a high prevalence in children exposed to ZIKV, ranging from 17.9% to 70%, as does reflux [118,120]. Santos et al. [115] showed in their study that 34.1% of children with congenital ZIKV syndrome had poor sleep quality, 3.5% had frequent nighttime awakenings, and 24% were able to sleep less than nine hours in a row.

ZIKV infection can also affect the heart of the child, most commonly in mothers infected in the second trimester of pregnancy (10%). Anomalies include atrial and ventricular septal defects, patent ductus arteriosus, cardiomyopathy, and hyperechogenicity of the aortic and mitral valves [115,117,118]. Pulmonary changes are usually present in more severe cases that progress to death. Autopsy findings include hydrothorax and diaphragmatic paralysis [118]. Urinary tract changes are seen in older children, including low bladder capacity, bladder hyperactivity, high bladder pressure during filling and high post-micturition residual volume, and recurrent urinary tract infections [118].

Finally, there is disagreement in the literature regarding the presence of neurodevelopmental delay in normocephalic children intrauterine infected with ZIKV. Currently, there are cognitive assessment tools that can be used at different times during childhood. According to Marbán-Castro et al. [119], the Bayley Scale of Infant and Toddler Development (BSID-III) was the most widely used, showing that about 30% of children had language delay, while 8.4% had moderate and severe delay. The study by Blackmon et al. [134] reported no evidence of cognitive or behavioral changes in normocephalic children exposed to intrauterine ZIKV. However, both studies concluded that the lack of control groups matched for socioeconomic and cultural conditions compromised the results of the research [119,138]. Further studies should be conducted to truly assess the neurocognitive changes in children exposed to intrauterine ZIKV in order to establish an appropriate segment. Table 3 shows the main postnatal findings of children exposed to intrauterine ZIKV.

## 10. Prognosis

The prognosis of children born during the 2016 ZIKV epidemic in Brazil remains an area of ongoing surveillance and research. The most severe sequela of infection, such as microcephaly, neurological malformations, and other complications associated with congenital ZIKV infection, continue to have a significant impact on the development of these children [139]. Although some progress has been made in terms of medical follow-up and interventions, the long-term prognosis of these children depends on a number of factors, including the severity of the infection, access to specialized care, and ongoing therapeutic interventions [139,140]. The impact on the cognitive development of children affected by congenital Zika infection can be profound and long-lasting. Many of these children have significant intellectual deficits, particularly those with microcephaly and other severe brain abnormalities. The cognitive prognosis depends on the severity of brain malformations, such as intracranial calcifications, brain atrophy, and cortical lesions, which are seen in many children [139,140,141].

It is possible to divide them into an unfavorable prognosis, characterized by children with severe microcephaly and extensive brain lesions, who tend to have a more severe prognosis with persistent difficulties in learning, remembering, and understanding concepts, and a more favorable prognosis, consisting of children with intrauterine ZIKV infection with moderate cognitive deficits [140,141]. It means that with appropriate interventions, such as specialized educational support and early stimulation therapy, children can achieve cognitive development milestones closer to those expected for their age group. Access to ongoing medical care, early intervention, and psychosocial support play a key role in improving the development of these children, while severe resource limitations in some areas may jeopardize the prognosis of many of them [140,141,142].

## 11. Short- and Long-Term Follow-Up

A study conducted between March and November 2016 found that the overall risk of adverse outcomes during pregnancy and early childhood, a critical period of life that affects social, educational, and health outcomes worldwide, was 15.7% [142]. It showed that in utero exposure to ZIKV was 15.7%, distributed as follows: 3.6% severe sequela or death; 2.7% major anomalies; and 9.4% minor anomalies [141]. The risk of severe sequela or death was higher when ZIKV infection occurred during the first trimester (7.0%) compared with the second (2.7%) or third (1.4%) trimesters [142,143].

The high incidence of ZIKV infection in Brazil in 2015–2016 may have affected the rate of linear height growth in children exposed to ZIKV in utero [143]. Seventy-one children born between March 2016 and June 2018 were followed up for anthropometric indices: z-score for body mass index, weight, height, head circumference, and growth velocity [143]. Four children had congenital microcephaly and severe neurological impairment. The other 67 were non-microcephalic children (60 normocephalic and 7 macrocephalic); of these, 24.2% (16 children) had neurological alterations, and 28.8% (19 children) had alterations in neuropsychomotor development. Seventeen (24.2%) children had low growth velocity. The frequencies of low growth velocity in microcephalic and non-microcephalic children were 25% (1 of 4 children) and 23.9% (16 of 67 children), respectively [113,142,144].

In another study, 35 children with congenital ZIKV syndrome were evaluated with the Bayley III scales at 12 and 24 months of age from November 2016 to December 2018 at a rehabilitation center in Brazil [142]. Inclusion criteria were children with an established diagnosis of congenital ZIKV syndrome. Exclusion criteria included the presence of arthrogryposis, prematurity, irregular follow-up, clinical complications, or other causes of microcephaly [140]. Children who developed cerebral palsy were classified according to the Gross Motor Function Classification System (GMFCS) at 2 years of age [139,140]. Congenital ZIKV infection was a risk factor for functional impairment in all domains of development, with a direct and substantial negative impact on early childhood development.

## 12. Conclusions

ZIKV infection is epidemiologically important because of the resulting adverse perinatal outcomes and the need for multidisciplinary care of the child for the rest of his/her life due to significant functional alterations with a predominance of cognitive deficits. For this reason, there is still concern about a new outbreak, since there are important challenges in terms of diagnosis, given the similarity of symptoms with other arboviruses and the cross-reaction with DENV, dengue virus, which has four different serotypes (DENV-1, DENV-2, DENV-3, and DENV-4) and can cause severe forms of dengue, including hemorrhagic fever and dengue shock syndrome. Both viruses are transmitted by *Aedes* mosquitoes, making differential diagnosis challenging.

Therefore, it is important to point out that after analyzing the data presented in this review, it is clear that although the main characteristic associated with ZIKV infection is microcephaly, there is evidence that other infected fetuses do not necessarily present this malformation but do exhibit physical and neurodevelopmental alterations that also affect the quality of life and survival of individuals, further emphasizing the need for prevention programs. Furthermore, there is a possibility of a lack of diagnosis, since after the multiple episodes of ZIKV infection, it has not become the focus of the main diagnostic hypothesis for a pregnant woman with flu-like symptoms, as other infections have emerged and become the new diagnostic target. For this reason, new preventive measures must be taken to avoid new epidemics of ZIKV, since this infection has a direct impact on the quality of life of the population and public health expenditure.

## Figures and Tables

**Table 1:** Main central nervous system abnormality findings in newborns with Zika virus intrauterine infection by computed tomography and magnetic resonance imaging.

Findings	Computed Tomography	Magnetic Resonance Imaging
Microcephaly	Reduced skull diameter and brain volume	Reduced skull diameter and brain volume
Cerebellar hypoplasia	It can be observed	It can be observed
Cortical abnormalities	Can identify volumetric reduction in the brain parenchyma	Best examination to identify cortical abnormalities, usually diffuse and predominantly in the frontal lobes
Prominence of the occipital bone	Detected	Detected
Intracranial calcifications	Sensitive to calcifications, especially in the subcortical junction, basal ganglia, thalamus, and brainstem	Detects calcifications but with less sensitivity compared to computed tomography
Ventriculomegaly and colpocephaly	Frequent	Can be identified
Enlargement of the extra-axial cerebrospinal fluid space	Possible to identify	Best test to identify enlargement, due to impaired reabsorption of cerebrospinal fluid caused by decreased brain volume
Changes to the skullcap	Everted sutures and prominent occipital bones	More detailed for changes such as the “collapsed” appearance of the skull

**Table 2:** Main prenatal differential diagnoses of Zika virus and other intrauterine infections.

	Zika Virus	Rubella	Cytomegalovirus	Toxoplasmosis
Gestational age period with higher risk of congenital infection	First trimester (between 7 and 13 weeks) [2]	First trimester (mainly in the 10th week) [2]	Mainly in the first trimester, but the risk remains in the second and third trimester [2]	Third quarter (especially in the last month of pregnancy) [132]
Adverse perinatal outcomes	Microcephaly and eye changes [3]	Sensorineural hearing loss, cataracts, cardiac anomalies, and neurological alterations such as microcephaly [2]	Sensorineural hearing loss, chorioretinitis, microcephaly, cerebral palsy, and intellectual disability [2]	Intracranial calcifications (50–80%), hydrocephalus, and chorioretinitis [132]
Brain calcifications	Predominantly in the subcortical region [131]	Periventricular and basal ganglia [131]	Periventricular [131]	-
Other ultrasound findings	Cerebral: ventriculomegaly, microcephaly; [133] Abdomen: no changes; Placenta: no changes; AFI: polyhydramnios; Limbs: arthrogryposis; Heart: no changes; FGR: yes [134]	Cerebral: meningocele, encephalocele, encephalitis, microcephaly; Abdomen: hepatosplenomegaly, intestinal hyperechogenicity; Heart: patent ductus arteriosus, pulmonary artery stenosis; Facial: micrognathia; Bone: bone radiolucency; FGR: yes [135]	Cerebral: ventriculomegaly, microcephaly; Abdomen: ascites, hepatic calcifications, hyperechogenic intestine, hepatosplenomegaly; Heart: pericardial effusion; Placenta: small or placentomegaly/placentitis; FGR: yes [136]	Cerebral: ventriculomegaly; Abdomen: hepatomegaly and hepatic calcifications; Placenta: thickened with normal echogenicity; Heart: pericardial effusion; FGR: yes [137]

AFI: amniotic fluid index; FGR: fetal growth restriction.

**Table 3:** Main postnatal findings of children exposed to intrauterine Zika virus.

Postnatal Findings	Signs/Symptoms
Central nervous system	Seizures Restless movements Hypertonia or hypotonia Irritability Hyperreflexia
Vision	Anterior and posterior segment alteration Microcornea Microphthalmia Circumscribed chorioretinal atrophy Retinal dysplasia Abnormalities in extrinsic motility Focal pigment spots Glaucoma Hypoplasia and excavation of the optic nerve Nystagmus
Hearing	Sensorineural hearing loss
Motricity	Arthrogryposis clubfoot Gross (cerebral palsy) and fine motor deficits
Oropharynx	Difficulty sucking Insufficiency of the orofacial muscles Dysphagia Reflux
Sleep–wake cycle	Poor sleep quality Night wakings
Cardiac	Septal defects (atrial and ventricular) Persistent cardiomyopathy Hyperechogenicity of the aortic and mitral valve
Pulmonary	Hydrothorax Diaphragmatic paralysis
Urinary tract	Reduced bladder capacity Bladder overactivity Increased bladder pressure in the filling phase Increased residual volume Recurrent urinary tract infections

## Data Availability

The data presented in this study are available on request from the corresponding author.
